# Exercise‐induced pain within endurance exercise settings: Definitions, measurement, mechanisms and potential interventions

**DOI:** 10.1113/EP091687

**Published:** 2024-07-10

**Authors:** Callum A. O'Malley, Samuel A. Smith, Alexis R. Mauger, Ryan Norbury

**Affiliations:** ^1^ School of Sport, Exercise, and Nutritional Sciences University of Exeter Exeter UK; ^2^ School of Sport and Exercise Sciences University of Kent Canterbury UK; ^3^ Faculty of Sport, Technology, and Health Sciences St Mary's University Twickenham UK

**Keywords:** aetiology, definitions, interventions, mechanisms, nociception, pain

## Abstract

**Abstract:**

Pain can be defined as an unpleasant sensory and emotional experience associated with or resembling that associated with actual or potential tissue damage. Though consistent with this definition, different types of pain result in different behavioural and psychophysiological responses. For example, the transient, non‐threatening, acute muscle pain element of exercise‐induced pain (EIP) is entirely different from other pain types like delayed onset muscle soreness, muscular injury or chronic pain. However, studies often conflate the definitions or assume parity between distinct pain types. Consequently, the mechanisms through which pain might impact exercise behaviour across different pain subcategories may be incorrectly assumed, which could lead to interventions or recommendations that are inappropriate. Therefore, this review aims to distinguish EIP from other subcategories of pain according to their aetiologies and characteristics, thereby providing an updated conceptual and operational definition of EIP. Secondly, the review will discuss the experimental pain models currently used across several research domains and their relevance to EIP with a focus on the neuro‐psychophysiological mechanisms of EIP and its effect on exercise behaviour and performance. Finally, the review will examine potential interventions to cope with the impact of EIP and support wider exercise benefits.

**Highlights:**

- **What is the topic of this review?**
  Considerations for future research focusing on exercise‐induced pain within endurance exercise settings.
- **What advances does it highlight?** An updated appraisal and guide of research concerning exercise‐induced pain and its impact on endurance task behaviour, particularly with reference to the aetiology, measurement, and manipulation of exercise‐induced pain.

## 1 INTRODUCTION

Pain is a ubiquitous phenomenon experienced across numerous everyday activities (Mense, 2003). Exercise is one activity which typically elicits an acute pain experience originating from the working musculature (Cook et al., 1997), herein referred to as exercise‐induced pain (EIP). This EIP is a common perception that affects individuals across the entire spectrum of exercisers, from sedentary individuals to elite athletes (Mense & Schiltenwolf, 2010). It is characterised as salient, intense and unpleasant, but is also both transient and non‐damaging (Cook et al., 1997). Therefore, EIP is often considered as something to ‘push‐through’ or ‘cope with’ to sustain exercise participation and enhance performance (Lasnier & Durand‐Bush, 2022).

The aim of this review is to provide an updated conceptual and operational definition of EIP, which has previously been conflated with other pain types and taxonomies (for example, delayed onset muscle soreness (DOMS) or exercise‐induced injury). This will be achieved by distinguishing between different subtypes of pain according to their aetiologies and characteristics. Subsequently, there will be a critical review of existing experimental pain models that have been used to understand EIP. Next, the review will discuss the neuro‐psychophysiological mechanisms that govern the effect pain has on exercise behaviour and performance. Finally, the review will conclude by outlining potential interventions that help individuals overcome EIP to sustain exercise participation and enhance exercise performance.

## 2 DEFINITION(S) OF PAIN

Pain, as defined by the International Association for the Study of Pain, is ‘an unpleasant sensory and emotional experience associated with, or resembling that associated with, actual or potential tissue damage’ (Raja et al., 2020). Therefore, pain is foremost a conscious phenomenon that arises from the complex integration of psychophysiological sensory signals (e.g., nociception) across the central nervous system (Almeida et al., 2004; Mense, 2009). Importantly, pain and nociception are independent entities (Julius & Basbaum, 2001). Nociception encompasses the sensory process of signalling noxious stimuli to the thalamic level, which can also be affected by descending projections from the periaqueductal grey and rostral ventromedial medulla (Rainville, 2002). Detailed discussion and review of the neurophysiology of pain and its modulation have been provided in many other excellent papers, to which we refer the reader (e.g., Almeida et al., 2004; Mense, 2003, 2009; Rainville, 2002; Rainville et al., 1992, 1997; Vadivelu et al., 2009). Meanwhile, pain is the product of the integration and comprehension of this stimulus into a perceived sensory and emotional experience (Julius & Basbaum, 2001; Raja et al., 2020). Additionally, the subjective pain experience can vary according to its duration (acute, chronic), anatomical location, aetiology and pathophysiology (nociceptive, neuropathic, inflammatory) thereby allowing researchers to subcategorise pain into several distinctive taxonomies/types (Thienhaus & Cole, 2002). Moreover, the experience of pain is highly sensitive to changes in sensory, affective, cognitive and motivational factors (Almeida et al., 2004), making it highly individualised (Mense, 2003). Therefore, it is theoretically plausible that an individual could be subject to identical sensory conditions (nociception) but the subsequent perception (pain) could differ due to various differences in sensory processing at pain‐related brain areas, as pain is dependent on a unique interplay between neurophysiological (Aboodarda et al., 2020) and socio‐cognitive factors (Craig & MacKenzie, 2021).

As a universally recognised perception, pain fulfils an important protective function by facilitating adaptive responses to maintain muscle function whilst minimising tissue damage (Hodges & Tucker, 2011; Vadivelu et al., 2009). During exercise, EIP constitutes one subcategory of the overall pain experience but features heavily in the self‐regulation of exercise behaviour (Venhorst et al., 2018). As a result, it is worthwhile operationally and conceptually distinguishing EIP from other common pain types that may also arise naturally (e.g., DOMS/injury‐related pain) in response to exercise‐based tasks.

### 2.1 Exercise‐induced pain

Relatively commonplace and well‐recognised within healthy populations when participating in exercise of a prolonged and intense nature (Cook et al., 1997), EIP manifests mainly from noxious, chemical nociceptive stimuli. Specifically, metabolites from anaerobic energy contributions such as potassium and hydrogen ions, substance P, histamines, prostaglandins, serotonin, bradykinin and adenosine within the intramuscular space stimulate free nerve endings supplied by C‐fibre, non‐myelinated group IV afferents (Graven‐Nielsen, 2006; Graven‐Nielsen & Mense, 2001; Mense, 1993; Pollak et al., 2014). Nociceptive signals are conveyed along group IV afferents via the dorsal horn of the spinal cord and then directed towards the thalamus (Basbaum et al., 2009), which discriminates the type of noxious stimuli (e.g., chemical, mechanical, thermal) and then relays nociceptive signals onto several cerebral areas (Basbaum et al., 2009). Accordingly, the insula and somatosensory cortex are thought to be involved with the comprehension of EIP *intensity* (Hofbauer et al., 2001). The integration of sensory nociceptive signals at the anterior cingulate cortex is believed to influence the *quality* and *affective* dimension of the EIP experience (Rainville et al., 1997). Whilst EIP is mainly a product of chemical nociceptive signals conveyed by group IV afferents (Pollak et al., 2014), mechanical (e.g., deformation of tissue increasing intramuscular pressures) nociception detected via Aδ, thin myelinated, group III muscle afferents and added thermal stimulation on group IV afferents also contribute to a lesser degree to the EIP experience (Graven‐Nielsen, 2006; Mense & Gerwin, 2010).

**FIGURE 1:**
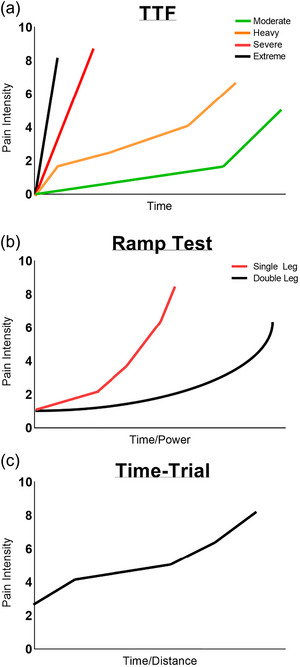
Typical pain intensity responses to different cycling exercise intensities and modalities. (a) Time to task failure (TTF) exercise during each intensity domain. Based on data from Ianetta et al. (2022). (b) Pain intensity during an incremental cycling ramp test using a single leg and double legs. Based on data from Zhang et al. (2021). (c) Pain intensity during a cycling time‐trial. Based on data from Mauger et al. (2010) and from Astorino et al. (2012).

Figure 1 demonstrates the typical EIP intensity ratings provided by healthy, active participants across varying intensities and modalities of prolonged exercise from prior studies. Cook et al. (1997) first identified that an EIP threshold tends to occur at the instance when muscle metabolite accumulation exceeds the clearance rate. In their studies, this intensity corresponded to approximately 50% peak power output during cycle ergometry (Cook et al., 1997). However, there are large inter‐individual differences in this threshold, with ranges reported from 25% to 95% of peak power output (Wender et al., 2023). Recent studies have evidenced that the development of EIP coincides closely with other physiological concepts such as the *critical intensity* (Iannetta et al., 2022), or gaseous exchange threshold (Burnley & Jones, 2018) that demarcates the boundary between moderate and heavy intensity exercise, which both involve predominantly aerobic metabolic contributions (Burnley & Jones, 2018; Iannetta et al., 2022). Furthermore, EIP is believed to increase linearly with time during efforts that remain within the severe or extreme intensity domains with predominant anaerobic metabolic contributions (Burnley & Jones, 2018; Iannetta et al., 2022; Smith et al., 2020). Conceptually, this is supported by the aetiology of EIP, whereby exercise above the *critical intensity* results in non‐steady state metabolic responses (Burnley & Jones, 2018), resulting in the accumulation of pain‐inducing metabolites (Pollak et al., 2014). Perceptions of EIP can still occur *below* the *critical intensity* (i.e., within the low or moderate intensity domains), due to modest concentrations of noxious biochemicals or allodynia‐type situations whereby the microenvironment in and around the muscle does not always reflect the cardiovascular demand of the task (Smith et al., 2019). Therefore, in some cases, there is a slight disconnect between the sensory information from the muscular environments and the perception of EIP (Smith et al., 2019). Importantly this can go in either direction, for example, there could be minor nociceptive stimulation but a more pronounced EIP response or more intense nociceptive stimulations but a disproportionately smaller EIP experience which may involve other factors such as endogenous pain modulations and/or exercise‐induced hypoalgesia (see Koltyn et al., 2014; Rice et al., 2019). For a further review on other sociological factors, neuropathic pain, and allodynia during exercise tasks, see Leitzeler and Koltyn (2021).

#### 2.1.1 Key characteristics of EIP

Several characteristics distinguish the EIP phenomenon from other exercise‐related pain experiences. Prior research has identified some of these major characteristics as:

- Pain originating within the working musculature involved with the exercise.
- An acute, tonic, transient pain experience that occurs *during* exercise.
- A non‐damaging pain that is a potential indicator of metabolic and/or cardiorespiratory demands of the exercise task.
- A pain phenomenon that the exerciser can control via behavioural (e.g., changes in exercise intensity) or other psychophysiological coping strategies.

As mentioned, whilst other pain types such as DOMS or exercise‐induced injury are ‘exercise‐induced’, they do not specifically correspond to the EIP definition and its core characteristics. To clarify, DOMS arises from unaccustomed, repeated (eccentric) muscular contractions that place muscle fibres under high levels of tension causing damage (Armstrong, 1984; Jubeau et al., 2012). In addition, DOMS and other injury‐related damage to muscles are associated with a release of algesic substances, muscle spasms and inflammation (Cheung et al., 2003). In contrast to EIP, pain associated with injury and DOMS involves a compilation of mechanical, thermal and chemical nociceptive and neuropathic stimulation (Cheung et al., 2003), culminating in a tonic pain experience (Julius & Basbaum, 2001) whilst there is recovery from the microinjuries from a prior exercise bout. Inability to recover presages the onset of chronic (>12 weeks) pain conditions that also are altogether distinct from EIP (see Mense & Gerwin, 2010).

Consequently, DOMS and injury exhibit three crucial differences from EIP. First, injury and DOMS involve a more long‐lasting pain experience that arises in the hours or days after exercise has been completed (Cheung et al., 2003). Second, injury and DOMS are indicative of *actual* damage to the muscle whereas EIP is a signalling of the intramuscular environment experiencing a short‐term perturbation from the resting homeostatic state which *could* result in damage if it remains unaddressed (Hodges & Tucker, 2011; Vadivelu et al., 2009). Third, DOMS is largely uncontrollable, especially during an exercise, as it is yet to manifest (Mizumura & Taguchi, 2024; Pollak et al., 2014).

Altogether, different pain types result in a unique pain experience. In turn, it is expected that exercise behaviour is adapted through varied central and peripheral mechanisms according to these different pain experiences (Hodges & Tucker, 2011). Subsequently, studies with an interest in pain‐related responses (Olesen et al., 2012; Staahl & Drewes, 2004) ought to consider the experimental pain models they use before drawing conclusions about how a specific pain type affects exercise behaviour.

### 2.2 Experimental pain models and their relevance to pain taxonomies

Experimental pain models provide a controlled and standardised means to activate the nociceptive system and evaluate the isolated behavioural, neurophysiological or psychophysiological responses to the evoked pain (Olesen et al., 2012; Staahl & Drewes, 2004). Whilst some studies provide a rationale for their chosen model, many do not consider the potential confounds of each model and the conclusions that are drawn from their data. Namely, each pain model results in varied processing of sensory signals and subsequent responses (Olesen et al., 2012). Thus, the selection of the pain model should be carefully considered to ensure that the nociceptive processing and elicited pain experience are relevant to the desired type of pain being investigated (Rainville et al., 1992).

A caveat of all pain models is that they do not *exactly* mimic EIP and therefore the outcomes of these methods (e.g., pain experience and subsequent motor behaviour) may not be directly comparable due to individual variances in tolerance thresholds or the perceived ability to adapt to specific pain types (Black, 2012). In equal measure, investigating EIP by observing naturally occurring EIP is also problematic as there is no plausible method within the domain of exercise in which a task can produce EIP without incurring other confounds on exercise behaviour such as fatigue, effort and affective changes (Aboodarda et al., 2020; Venhorst et al., 2018). However, it can be argued that certain pain models stimulate similar pathways and/or elicit pain qualities and potential motor adaptations that are reflective of a true EIP experience (Rainville et al., 1992). Therefore, the rationale for using pain models is to impose pain experiences that may have similarities with EIP or exacerbate existing pain during an exercise task and thereby disaggregate experimental pain from other exercise‐related phenomena to ascertain its role in motor behaviour. Consequently, this section gives a short summary of the pain pathways of each of these methods in comparison to EIP and the subsequent pain experiences and effects on motor behaviour. As such, this section will highlight some of the most prevalent experimental pain models used within the current literature and identify the appropriateness or shortcomings of each model to understand the mechanisms and effects of EIP. This list is not exhaustive and for a comprehensive review of pain models, the reader is directed towards Olesen et al. (2012).

#### 2.2.1 Hypertonic saline injections

Hypertonic saline pain induction models typically consist of an infusion of a small (0.5–1.5 mL) bolus of 5%–6% sodium chloride into a muscle belly, predominantly acting to stimulate group IV nociceptors (Graven‐Nielsen, 2006). Nociceptive stimulation is thought to occur potentially through membrane depolarisation by hydrogen ions (Graven‐Nielsen, McArdle, et al., 1997; Mense, 2009) or indirectly through glutamate release (Tegeder et al., 2002). Thus, hypertonic saline models closely mimic the natural EIP nociceptive pathways (Graven‐Nielsen, 2006).

There are several benefits of the hypertonic saline model for researchers interested in EIP. First, hypertonic saline can be standardised according to its location, volume and time course (Smith et al., 2023), thus affording researchers the ability to elicit an *artificial* EIP response that reflects the transient and non‐toxic/non‐damaging nature of *natural* EIP (Graven‐Nielsen, 2006; Smith et al., 2020). Second, the hypertonic saline model can include an accompanying procedural matched control condition using isotonic saline infusions of ∼0.9% sodium chloride (Graven‐Nielsen et al., 2002). Third, hypertonic saline does not interfere with the electrophysiological properties of the muscle (Farina et al., 2005) meaning it can be administered at the beginning of an exercise without any confounding effects on muscular contractile properties in effect (Graven‐Nielsen et al., 2002). Finally, studies show that the hypertonic saline model is reliable on the intra‐individual level (Smith et al., 2023) at effectively replicating the qualitative experience of naturally occurring EIP (Smith et al., 2020).

However, there are some potential drawbacks to the model. A potential issue with the model is that it is difficult to homogenise the experimental pain response (Graven‐Nielsen, Svensson, et al., 1997). Unlike quantitative sensory testing, some within‐subject and inter‐individual designs have shown varied pain responses (Smith et al., 2023, 2021). However, it is hard to judge why the responses can be different on an intra‐ and inter‐individual basis. One solution could be an individualised volume of saline administration according to anatomical characteristics or information based on previous pain exposure (Smith et al., 2023), yet it is unlikely a completely uniform experimental pain response would be possible unless a continual infusion took place, which may be unfeasible if intense muscle contractions need to be performed in the same limb. Altogether, whilst the hypertonic saline model does have some limitations, overarchingly, research has found it to be a valid and acceptably reliable means of experimentally inducing EIP‐like experiences to explore the subsequent behavioural and psychophysiological responses during exercise (Graven‐Nielsen, Arendt‐Nielsen, et al., 1997; Smith et al., 2023). 

### 2.3 Blood flow restriction and cuff algometry

**FIGURE 2:**
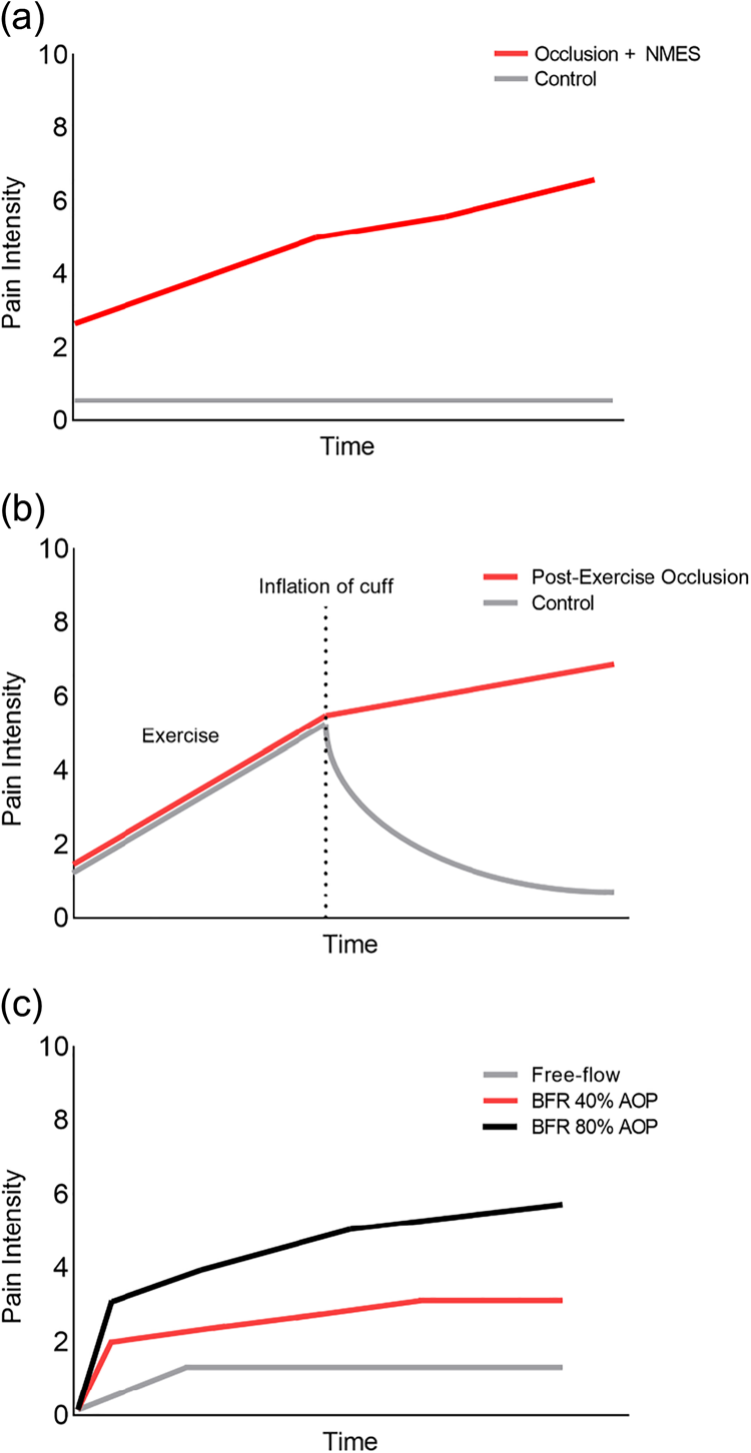
Pain responses to (a) neuromuscular electrical stimulation combined with blood flow restriction. Based on data from Zhang et al. (2023). (b) Post‐exercise circulatory occlusion. Based on data from Finn et al. (2018). (c) Moderate intensity cycling (40% ${\dot{V}}_{O_{2}\max}$) exercise at low (40%) and high (80%) arterial occlusion pressures. Based on data from Hughes et al. (2021). Abbreviations: AOP, arterial occlusion pressure; NMES, neuromuscular electrical stimulation.

Another commonly used method to induce EIP‐like experiences involves the inflation of a pneumatic tourniquet around the limb(s). Prolonged inflation (1–10 min) of a tourniquet at or above the arterial occlusion pressure can induce low to moderate perceptions of pain at rest (Norbury et al., 2023) with compressive forces likely stimulating group III afferents of the underlying skin and musculature (Patterson et al., 2019). Furthermore, restriction of venous return, precludes metabolite clearance from working muscles causing stimulation of group IV afferents, especially with prolonged periods of occlusion, or concurrent motor activity and blood flow restriction (Aboodarda et al., 2020). In recent studies (see Figure 2), occlusion of a contralateral, non‐exercising limb resulted in an inexorable rise of muscle pain to (near) maximal levels (Aboodarda et al., 2020; Azevedo de Almeida et al., 2022). Neuromuscular electrical stimulation of the occluded limb has also been used to exacerbate pain without the need for voluntary motor output (Zhang et al., 2023). However, perhaps the most frequently used method of inducing experimental pain or exacerbating EIP is with the performance of voluntary muscle contractions during tourniquet inflation (McClean et al., 2023). The intensity of pain through this method can be altered by manipulating tourniquet pressure, with greater pressures causing greater pain (Patterson et al., 2019). Typically, 40%–80% of arterial occlusion pressure is used during resistance and aerobic exercise at low contraction intensities of <50% of 1 repetition maximum/${\dot{V}}_{O_{2}\max}$ (Patterson et al., 2019). Alternatively, perceptions of EIP can be induced with free‐flow exercise, then pain intensities can be maintained by inflation of the tourniquet to high pressures, often referred to as ‘post‐exercise circulatory muscle occlusion’ (Zambolin et al., 2023).

Evidently, the use of tourniquets can provide a versatile and robust means to induce experimental pain or exacerbate pre‐existing EIP. Furthermore, the manipulation of tourniquet pressure and/or exercise variables provides a means to regulate the intensity and duration of EIP to desired amounts. Given the benefits and accessibility of this method, it can be an attractive model that suitably investigates EIP. In particular, blood flow restriction can be useful for investigating when pain is not localised to the exercising site (e.g., contralateral limb) or the residual effects of pain on subsequent exercise behaviour, though the same can be said for most pain models too. However, there are some limitations to blood flow restriction. Chiefly, if the induction of pain and the subsequent assessment of physiological responses are performed on the same limb, then findings will be severely confounded by neuromuscular fatigue induced by localised hypoxia (Goodall et al., 2010). Additionally, it may be that some of the pain induced by the tourniquet (i.e., compressive forces) likely arises from stimulation of cutaneous nociceptors (Olesen et al., 2012). Lastly, unlike hypertonic saline infusions which invoke an immediate pain response (Graven‐Nielsen, 2002), the ischaemic component of blood flow restriction is not immediate and often increases with the intensity and duration of the exercise (Patterson et al., 2019). Thus, blood flow restriction may be susceptible to an exercise‐induced hypoalgesia effect whereby endogenous pain modulation of two pain sources (exercise and blood flow restriction) could impact the overall pain experience of interest. Therefore, blood flow restriction may elicit some experiences not truly reflective of the EIP phenomenon as well as some potential confounding interactions, meaning it may be less suitable as an experimental model for investigating EIP compared to other approaches.

### 2.4 Thermal stimulation

Acute temperature changes at the skin or other peripheral organs over 43°C or below 15°C evoke a thermal nociceptive sensation via group III and IV afferents (Tominaga & Caterina, 2004). Within experimental studies, several devices such as quantitative sensory testing systems have been applied to the skin during a motor task to assess the effects of specific thermal stimulations on the pain experience and subsequent behaviour (e.g., Dancey et al., 2016; Gandhi et al., 2013). Additional methods such as capsaicin cream can also influence the thermal pain sensation by triggering an earlier opening of thermosensitive ion channels to stimulate thermal pain perceptions at more temperate (between 15°C and 43°C) thermal exposures without actual tissue damage (Dancey et al., 2016). 

Indeed, EIP does have some minor thermal nociceptive contributions due to natural increases in muscle temperature during exercise activity (Mense, 1993). Therefore, there are some crossovers between some of the subjective experiences between EIP and thermal pain like pain feeling ‘hot’ or ‘burning’ (Cook et al., 1997). In addition, thermal pain stimulation via quantitative sensory testing can be closely controlled by an experimenter to elicit specific pain intensities in a standardised manner. For example, participants can undergo a calibration to thermal pain by applying different temperatures across sites of interest. Participants can provide a continual pain intensity rating which can be plotted against the temperatures to deduce which temperatures elicit specific pain intensities. Furthermore, additional calibrations can be performed at certain temperatures to ascertain whether temporal or other contextual factors (e.g., completing a task) may affect the pain rating. As such, thermal stimulation techniques offer a controllable method to induce specific pain intensities which imitate some aspects of the EIP experience. 

However, there are some considerations if using a thermal pain model. Foremost, the thermal element of EIP is internal whereas thermal pain models are cutaneous. Additionally, EIP and thermal pain have disparate affective/motivational and evaluative effects that stem from different neuronal processing of pain qualities within the brain (Hofbauer et al., 2001) or varied metacognitive consequences about the pain experience (i.e., the individual being consciously aware that the pain they are experiencing is from a hot/cold device versus exercise). As a result, it is likely that thermal pain stimulation and EIP result in different behavioural responses during goal‐directed tasks like exercise. For instance, Wilcox et al. (2018) indicate that painful perceptions associated with heat stimulation are expected to have distinct cognitive‐evaluative processes compared to EIP‐type experiences. Namely, EIP is considered non‐damaging whereas thermal pain can pose an immediate risk to the tissues which can compel an individual to adopt more overt withdrawal‐type behaviours (Venhorst et al., 2018). Another consideration is that repeated thermal stimulations result in temporal summation due to a sensitisation of thermal nociceptors at regions where thermal stimulations have been applied (Dancey et al., 2016), meaning that although an experimenter could apply a continual thermal stimulation, the pain intensity responses may increase.

Therefore, whilst thermal pain models offer a standardised approach to elicit pain perceptions, the appropriateness of the thermal pain that the models evoke may be questionable in relation to the EIP phenomenon (Rainville et al., 1997; Wilcox et al., 2018). Bearing this in mind, researchers may want to consider the focus of their studies to help decide whether a more standardised pain delivery via thermal stimulations or more ecologically relevant alternatives (e.g., hypertonic saline/blood flow restriction) may be more appropriate. 

### 2.5 Electrical stimulation

Pain models using stand‐alone electric stimulations, or stimulations in conjunction with other pain models like blood flow restriction to facilitate the inducement of pain, are also available (Zhang et al., 2023). Electrical stimulations involve the application of small electrical currents to a skin surface which penetrate towards deeper tissues. The depth and intensity of pain from these currents can depend on the intensity of stimulation(s), location on the body, and equipment used. For instance, high‐frequency stimulations penetrate into deep tissues producing heat‐like effects whereas low‐frequency stimulations spread across superficial tissues and result in a more ‘smarting’ pain experience (Laursen et al., 1997). Electrical stimulation methods can be applied in an ‘on’ or ‘off’ fashion (Laursen et al., 1997) whereby researchers can provide transient and periodic stimulation to skin sites or more continual stimulations. However, low‐frequency electrical stimuli can inadvertently excite motor neurons causing muscle twitch. Moreover, in a similar manner to thermal stimulations, when applied repeatedly or at an increased intensity, this method can induce temporal summation resulting in a disproportionate increase in pain intensity and locality of the pain experience, thus demonstrating how electrical stimulation may cause central changes that EIP may not (Schulte et al., 2004).

Pain from electric stimulation methods is dissimilar in quality to EIP as it is often described as ‘boring’ and ‘penetrating’ with no long‐lasting effects (Laursen et al., 1997). In contrast to EIP, electrical stimulation is a non‐physiological method of pain induction that circumvents the nociceptors and directly facilitates both nociceptive and non‐nociceptive afferent input (Graven‐Nielsen & Mense, 2001; Olesen et al., 2012; Staahl & Drewes, 2004). In addition, electrical stimulation can sometimes evoke a concurrent muscle twitch, confounding the produced sensations of pain and causing issues with reproducibility (Graven‐Nielsen & Arendt‐Nielsen, 2003). As a result, electrical stimulations are not the most applicable when trying to mimic the EIP experience, but they may be suited to other pain taxonomies or as a facilitator of pain when used in conjunction with other experimental pain models. 

## 3 MEASUREMENT OF PAIN

Most recent studies (e.g., Canestri et al., 2021; Norbury et al., 2022a, 2022b; Smith et al., 2020, 2021) have adopted a combination of unidimensional and multidimensional self‐report scales to capture the intensity and qualities of EIP. For example, visual analogue scales (VAS) typically measure EIP intensity (the sensory dimension) on a scale of 0–10 or 0–100 and are commonly anchored by verbal descriptors ranging from ‘no pain’ to either ‘worst imaginable pain’ (VAS and numerical rating scale), or ‘severe pain’ (verbal rating scale) or ‘the most intense pain imaginable’ ​(Hawker et al., 2011)​. These scales are generally administered in verbal or written form, although scales such as the VAS are translatable to electrical devices to continuously record pain over time. However, pain intensity as the focal measure of pain only provides a singular classification of the overall experience, and therefore additional scales can facilitate the identification of other dimensions such as affect ​(Rainville et al., 1992) or soreness (Svensson & Arendt‐Nielsen, 1995).

Consequently, additional scales such as the McGill pain questionnaire (Melzack, 1975, 1987) address several elements and components (e.g., location, quality) beyond the magnitude of EIP intensity and consider it in a multidimensional context ​(Katz & Melzack, 2011; Melzack & Casey, 1968). Used to outline the ‘language of pain’, the McGill pain questionnaire defines the sensory, affective, evaluative and miscellaneous classifications of pain alongside an indication of overall pain intensity and distribution (Melzack, 1975). A short‐form version (Melzack, 1987) also exists containing a reduced number of words describing the sensory and affective classifications. The short‐form version provides a more rapid assessment of pain experiences like EIP as there are potential issues with longer assessments with poorer recall and the need to prioritise other protocol‐based requirements (Melzack, 1987). In summary, a multidimensional approach can provide a more holistic understanding of the EIP experience compared to standalone unidimensional measures of intensity (Katz & Melzack, 2011). However, there must be consideration for whether longer questionnaires such as the McGill pain questionnaire can be incorporated into the research design without detracting from the main research question. Furthermore, measurement techniques beyond kinesiology research have also advanced, such as the use of quantitative sensory testing, which compared to pain rating assessments provide a specific measure of the body's response to an internal/external stimulus rather than a general experience of pain denoted by a number.

### 3.1 The impact of EIP on endurance exercise performance

Few studies have explored how pain tolerance specific to EIP (i.e., the maximum level or time that someone is able to withstand EIP) can impact exercise performance (Black, 2012; O'Connor & Cook, 1999). Astokorki and Mauger (2017a) observed that when undertaking a cycling exercise at a fixed rating of perceived exertion, EIP tolerance, particularly the time one can withstand a submaximal intensity of EIP, can account for 7.5% of variance in endurance performance. As such, it is suggested that the ability or willingness to tolerate and/or moderate EIP could be a key differentiating factor in successful performance between individuals with a similar physiological capacity ​(Astokorki & Mauger, 2017a; Black, 2012; Cook et al., 1997; O'Connor & Cook, 1999)​. For specificity, this review will highlight studies that focus on EIP or experimental pain aimed at mimicking EIP‐type experiences and their relation to endurance‐based exercise performance in which endurance exercise encompasses activities with repeated muscular contractions for a prolonged period (>75 s).

Wender et al. (2023) recently identified a net negative effect of EIP on exercise performance. That is, as EIP is present or increases, exercise performance is impaired compared to instances where EIP is absent or lower. These investigations involve naturally occurring EIP (e.g., Cook et al., 1997) or experimentally induced pain mimicking EIP at local (e.g., Norbury et al., 2022a) or non‐local sites (e.g., Norbury et al., 2022b) via some of the abovementioned pain models.

Of the published literature, the most common way EIP‐like experiences have been increased during exercise is through an intramuscular injection of hypertonic saline. Results from these studies have consistently found that when hypertonic saline is injected into the muscle shortly before the performance of endurance exercise, pain is exacerbated and time‐to‐task failure is shortened in comparison to a non‐painful, isotonic saline condition ​(Canestri et al., 2021; Ciubotariu et al., 2004; Graven‐Nielsen, Svensson, et al., 1997; Norbury et al., 2022a; Smith et al., 2020)​​. This observation has been seen across a range of exercise intensities, modalities and muscle groups (Table 1). In fact, only one study has failed to observe a decrease in endurance performance ​(Schulte et al., 2004),​ but the null findings may be explained by a low peak pain response caused by the hypertonic saline (3.2/10) and the endurance task only starting once pain had peaked, meaning that the clearance of hypertonic saline (and pain) likely occurred before task failure was reached (Smith et al., 2020) or potentially that exercise‐induced hypoalgesia effects may have counteracted the relatively low peak pain response observed in the study (Rice et al., 2019). 

**TABLE 1:** Summary of the literature on the effects of localised experimental muscle by hypertonic saline injections on endurance performance.

Reference	Experimental pain stimulus	EIP intensities between conditions (au)	TTF exercise	Change in TTF compared to control saline (%)
Canestri et al. (2021)	Hypertonic saline, 2 mL, 6% NaCl, vastus lateralis	∼4.5 in painful vs. ∼3.5 in control	80% *W*_peak_ cycling	↓ 16.9^*^
Ciubotariu et al. (2004)	Hypertonic saline, 1 mL, 6% NaCl, tibialis anterior	6.3 in pain, no pain values reported in control	50% MVT dorsiflexion isometric contraction	↓ 10.0*
	Hypertonic saline, 1 mL, 6% NaCl, gastrocnemius	6.5 in pain, no pain values reported in control	50% MVT plantarflexion isometric contraction	↓ 9.9*
	Hypertonic saline, 1 mL, 6% NaCl, gastrocnemius	6.1 in pain, no pain values reported in control	80% MVT plantarflexion isometric contraction	↓ 25.5*
Norbury et al. (2022a)	Hypertonic saline, 1 mL, 5.85% NaCl, vastus lateralis	5.7 in painful vs. 3.8 in control	∼20% MVT knee extension isometric contraction	↓ 16.2*
​​Schulte et al. (2004)​	Hypertonic saline, 1 mL, 5.8% NaCl, biceps brachii	3.2 in pain, no pain values reported in control	40% MVT elbow flexion isometric contraction	ns ⟷
Smith et al. (2020)	Hypertonic Saline, 1 mL, 5.85% NaCl, vastus lateralis	6.3 in painful vs. 5.5 control	10% MVT knee extension isometric contraction	↓ 26.0*

* Statistically significant compared to a control condition (*P *< 0.05). Abbreviations: EIP, exercise‐induced pain; MVT, maximum voluntary torque; ns, non‐significant; TTF, time to task failure; *W*_peak_, peak power output.

Interestingly, the negative effects of experimental pain that mimic EIP also occur when the pain is present in non‐exercising muscles. Separate studies utilising either blood flow occlusion (Aboodarda et al., 2020; Azevedo de Almeida et al., 2022) or hypertonic saline (Norbury et al., 2022b) in one leg have observed a decrease in time to task failure in the contralateral leg. However, the effect seems to be less prominent than with localised experimental pain or EIP, as identical experimental pain stimuli result in less of a decline in endurance performance with contralateral (∼10%) versus local pain (∼16%), despite greater mean pain differences with non‐local pain (Norbury et al., 2022a, 2022b). However, studies directly comparing the effects of local versus remote pain on endurance performance are required to confirm this. To add, the kinetics of experimental pain should be an important factor in experimental design. With hypertonic saline injections, peak pain occurs relatively quickly (∼45–60 s), then slowly declines over the course of several minutes (Norbury et al., 2022b; Smith et al., 2023) whereas with the blood flow restriction models, EIP slowly rises to near maximal levels (Aboodarda et al., 2020). As a result, certain models that evoke slower pain responses may be subject to different interacting effects such as central pain modulation compared to fast‐acting models (Koltyn et al., 2014). A summary of recent research findings concerning endurance performance and EIP‐like pain at non‐local sites can be seen in Table 2.

**TABLE 2:** Summary of the literature on the effects of non‐local experimental muscle pain on endurance performance.

Reference	Experimental pain stimulus	EIP intensities between conditions (au)	TTF exercise	Change in TTF compared to control (%)
Aboodarda et al. (2020)	Resting blood flow occlusion, contralateral leg	∼ 6.9 in painful vs. assumed 0 in control	80% *W*_peak_ unilateral cycling	↓ 20.7^*^
Norbury et al. (2022b)	Hypertonic saline, 1 mL, 5.85% NaCl, contralateral vastus lateralis	3.3 in painful vs. 0.4 in control	∼20% MVT knee extension isometric contraction	↓ 9.8*
Azevedo de Almeida et al. (2022)	Resting blood flow occlusion after single‐leg cycling, contralateral leg	∼ 9 in painful vs. ∼ 0 in control	25% MVT knee extension isometric contraction	↓ 52.1*
Sandbach et al. (2022)	Resting blood flow occlusion after elbow flexion exercise	5.8 in painful vs. 4.5 in control	30% 1RM elbow flexion exercise	ns ⟷
Zhang et al. (2023)	Blood flow occlusion combined with neuromuscular electrical stimulation	∼4.6 in painful vs. ∼0.2 in control	30%–40% MVT intermittent isometric contraction of knee extensors	↓ 12.1*

* Statistically significant compared to a control condition (*P *< 0.05). Abbreviations: EIP, exercise‐induced pain; MVT, maximum voluntary torque; ns, non‐significant; RM, repetition maximum; TTF, time to task failure; *W*_peak_, peak power output.

In summary, there is a considerable body of evidence that indicates increased EIP, or experimental pain that mimics EIP, at exercising and non‐exercising muscles reduces endurance performance. However, the related studies involved time‐to‐task failure whereby pain affects the individual's ability to continue the task despite external task demands remaining constant. Very few studies seem to have investigated the effect of EIP during submaximal or self‐paced exercise performance, thereby limiting the generalisability of existing findings to exercise modalities where individuals are able to continually adjust exercise intensity. In addition, there are few studies that show EIP reaching maximal levels at task failure (Iannetta et al., 2022; Staiano et al., 2018) and for that reason some have suggested that it is not EIP that causes the decision to stop exercise. Rather, it is suggested that EIP may influence endurance performance by acting through several, indirect mechanisms, which will be addressed below.

## 4 MECHANISMS: HOW PAIN IMPACTS ENDURANCE PERFORMANCE

It is commonly accepted that the experience of EIP during repeated, prolonged muscular contractions is often associated with changes in motor behaviour with the fundamental purpose of those changes being to minimise further tissue damage or injury by alleviating the load on the painful tissue and reducing the existing perception of pain ​(Wender et al., 2023)​. Typically, behavioural changes involve reductions in exercise intensity (Mauger, 2013). Whilst such behavioural changes are immediately beneficial at limiting potential effects of EIP and reducing its intensity, they come at the expense of inferior task performance and a lower likelihood of goal attainment (Venhorst et al., 2018). Therefore, a better understanding of the mechanisms behind EIP's effect on exercise can provide individuals with the knowledge of suitable ways to cope or deal with EIP without compromising exercise goals.

### 4.1 Neurophysiological mechanisms

To elucidate mechanisms which underpin a reduction in endurance performance with EIP, experimental work has employed various neurophysiological techniques to assess changes at the cortical, spinal, and peripheral level during painful experiences. Notable methods include surface and intramuscular electromyography (EMG), peripheral nerve stimulation and transcranial magnetic stimulation (TMS). Most studies have used surface EMG to examine muscle activation levels during isometric contractions in response to EIP or experimental pain. Findings from early studies (and more recent work) indicate no change (Schulte et al., 2004; Norbury et al., 2022a; Smith et al., 2020) or a decrease in agonist activity (Ciubotariu et al., 2004, Farina et al., 2005) compared to non‐painful, force matched conditions. This is perhaps due to the limited detail global EMG amplitudes can offer about motor control strategies (Farina & Gallina, 2020). Furthermore, findings between studies are further complicated by the impact of different muscles, and their functional relevance (quadriceps, locomotor vs. adductor pollicis, fine motor control) and contraction intensities (high vs. low force), highlighting the need for more research replicating and comparing previous methods. More intricate techniques have been utilised to gain greater insight into the effect of EIP on motor control, and to provide some clarity on previous findings. For example, Farina et al. (2004) utilised intramuscular EMG, to record firing rates of individual motor units in response to experimental pain induced by hypertonic saline. They found that during pain, motor units decreased in firing frequency, with a greater pain intensity causing a greater reduction in firing rate, supporting the notion that pain inhibits muscle activity, which contrasts with the findings of studies measuring surface EMG amplitudes. However, despite reduced motor unit activity in these studies, voluntary torque production is maintained at the desired target level, which implies that there are central/peripheral compensatory mechanisms which allow for maintenance of force. 

A decrease in recruitment threshold and increase in firing rates of non‐painful synergist muscles could be a potential source of compensation, but this is unlikely, as previous work has demonstrated that motor unit firing rates in synergist muscles also decrease in response to pain from hypertonic saline injections (Hodges & Tucker, 2011), and this has also been observed in non‐painful antagonist/distal muscle (Cleary et al., 2022). Peripheral adjustments to motor units are also possible, but evidence does not support this (Farina et al., 2004; Norbury et al., 2022a). Therefore, the most probable explanation is the alteration and/or redistribution of muscle activity *within* the painful muscle(s) to maintain force production. Indeed, evidence to support this mechanism comes from initial work where EIP‐like pain induced by hypertonic saline injections resulted in the recruitment of new motor units, which was not expected based on the recruitment order observed during higher force, non‐painful contractions (Tucker et al., 2009). Furthermore, recent work utilising high‐density surface EMG during painful contractions at low (20% of maximum force) and high intensities (70% maximum force) has revealed divergent responses in high and low threshold motor unit behaviour, with a reduction in low‐threshold motor firing frequency, and a reduction in the recruitment threshold of high‐threshold motor units ​(Martinez‐Valdes et al., 2020).​ In short, high threshold motor units are recruited at lower forces to maintain task demands. Spatial activation patterns revealed with high‐density surface EMG also change in response to pain, with data suggesting that when hypertonic saline is injected into the muscle, the redistribution of muscle activity is impaired, which is an important functional adaptation to maintain task performance in the presence of neuromuscular fatigue (Falla & Gallina, 2020). Taken together, studies recording muscle and motor unit activity during EIP suggest that there is a centrally mediated alteration in motor control strategies which allows for the maintenance of task performance in the presence of EIP.

In further support of a central effect of EIP, evoked responses from TMS indicate decreased excitability along the corticospinal pathway (Chowdhury et al., 2022). In particular, Norbury et al. ​(2022a)​ discerned that increases in pain from an intramuscular hypertonic saline injection caused a significantly greater increase in the TMS silent period (reflecting corticospinal inhibition) during exercise, compared to an isotonic (non‐painful) injection, although this has not been consistently observed when the pain was in the contralateral limb ​(Azevedo de Almeida et al., 2022; Norbury et al., 2022b). Furthermore, muscle pain has also been shown to increase short interval intracortical inhibition but only after pain had resolved ​(Schabrun & Hodges, 2012). The silent period is thought to reflect GABA_b_ activity whereas short interval intracortical inhibition is thought to indirectly represent GABA_a_ activity, indicating a potential role of GABA‐mediated inhibition. Furthermore, peripheral nerve stimulation during and after maximal voluntary isometric contractions reveals that voluntary activation of muscles is diminished during EIP‐like pain, whereas potentiated twitch torque is unaffected (Norbury et al., 2022a). More research is needed to confirm these findings, however.

The consequences of these neural adjustments have clear and significant negative implications for endurance exercise performance. Firstly, given that higher‐threshold motor units are more prone to fatigue than their lower threshold counterparts (Burke, 1980), the performance of endurance exercise in the presence of pain may contribute to faster performance fatigability, due to a reduced contribution of fatigue‐resistant motor units (Martinez‐Valdes et al., 2020). Furthermore, because some motor units are inhibited, the ability to produce maximal forces is compromised. Increased EIP‐like pain from the injection of hypertonic saline has consistently been shown to reduce the maximal force generating capacity of the muscle ​(Graven‐Nielsen et al., 2002; Norbury et al., 2022a)​, and the decreases in maximum strength will make an absolute exercise intensity a greater relative intensity. For example, Graven Nielsen et al. ​ (2002)​ demonstrated that maximum voluntary torque decreased by 21% in the knee extensors after an intramuscular injection of hypertonic saline, which would change a 20% (pain free) maximum voluntary torque task to 25% of the maximum. Therefore, an increase in the relative exercise intensity in the presence of pain may also be responsible for reducing endurance time.

### 4.2 Psychophysiological mechanisms

Psychological factors do not operate independently and likely function in connection with neurophysiological changes, hence the phrase ‘psychophysiology’ used in this review. Subsequently, in tandem with neurophysiological mechanisms (e.g., motor‐corticospinal changes) that were previously outlined, EIP may also have some distinctive psychological consequences (Rainville et al., 1997), which may provide more context to how an individual completes an exercise task differently when experiencing EIP or experimental pain that mimics EIP (Venhorst et al., 2018). 

Foremost, when EIP is rated at a high intensity due to engagement in more vigorous exercise or from a more intense experimental stimulation of afferents (Iannetta et al., 2022), it is usually viewed as unpleasant and can cause distress (Venhorst et al., 2018). Furthermore, EIP is also thought to effect strong changes on an individual's motivation for a task (Mauger, 2013). Collectively, the aversive nature of EIP is thought to reduce the perceived benefit of the exercise task as one must withstand unpleasant sensations to reach the desired goal (Vogel et al., 2020). Indeed, Taylor et al. (2022) recently identified that an individual's performance goal value and their desire to continue investing effort into the task reduces as one transitions through moderate, heavy and severe exercise intensities wherein EIP is expected to increase in a linear fashion (O'Connor & Cook, 1999). Therefore, EIP is a strong motivator for individuals to decide to retract from activities that perpetuate the painful experience (Vadivelu et al., 2009) resulting in poorer exercise performance. Specifically, EIP likely reduces the subjective value of continuing an exercise task, thus making it less likely for an individual to continue to invest their resources to perform at a better standard in the presence of EIP (Vogel et al., 2020).

Another psychophysiological consequence of EIP is that if individuals wish to persist with an exercise aiming at their goal, they must continually override the natural protective response associated with pain perceptions such as discontinuing the exercise or reducing exercise intensity (Cook et al., 1997; Vadivelu et al., 2009). This phenomenon is referred to as response inhibition (Englert et al., 2021) and repeated response inhibition is thought to impose a motivationally fatiguing effect (Müller & Apps, 2019). Several studies have exhibited that cortical areas associated with inhibitory control (e.g., anterior cingulate cortex) demonstrate an increased activation when experiencing pain (Hofbauer et al., 2001; Rainville et al., 1997). As a result, over a prolonged exercise bout with EIP, an individual is more likely to be susceptible to increased perceived fatigability due to the repeated requirement to quell natural responses to withdraw from pain versus instances without pain when less response inhibition is required (Englert et al., 2021). 

However, in some instances EIP can be low to moderate in intensity during exercise, and in which, EIP may not always be detrimental to task performance (Gandhi et al., 2013). For example, Torta et al. (2017) demonstrate that attending to pain‐related stimuli can cause a displacement in attention from other perceptions like effort. Consequently, if EIP perceptions are at a level that is detectable but not overwhelming (e.g., low‐moderate), this pain may potentially prevent other exercise‐limiting phenomena like effort becoming too high (Torta et al., 2017). Comparable evidence from studies with experimental pain has shown that during low pain intensities from thermal stimulations, painful conditions may increase motivational drive to obtain a reward and therefore improve task performance (Gandhi et al., 2013). Therefore, whilst most of the studies that have been related thus far demonstrate a negative effect of EIP on exercise performance, there may be some indicators that an individual can still control some factors involved with the pain experience (especially when pain/EIP is at a tolerable/lower level) to maintain or even enhance performance (Gandhi et al., 2013; Lasnier & Durand‐Bush, 2022; Vogel et al., 2020). Consequently, this review will conclude with some possible interventions to overcome EIP during exercise and potentially enhance performance.

## 5 ACUTE INTERVENTIONS TO REDUCE EIP DURING ENDURANCE EXERCISE

Generally, the presence of EIP has a predominantly negative impact on endurance performance through established mechanisms. Therefore, individuals may seek to improve endurance exercise performance by controlling or limiting their EIP and other pain‐related responses. Subsequently, there are two primary ways an individual could influence their EIP experiences. One is to alter the nociceptive signalling via epidurals or other pharmacological interventions, thus blocking sensory information from reaching the brain (Mauger, 2013). Yet, this approach often causes carryover effects on other perceptions and may pose ethical concerns (e.g., banned substances). Alternatively, implementing interventions which can cause hypoalgesia via changes in the processing of nociceptive signals and therefore help an individual to cope or manage with EIP may be more appealing (Lasnier & Durand‐Bush, 2022).

### 5.1 Caffeine

Perhaps one of the most widely used ergogenic aids in sport and exercise is caffeine. Indeed, whilst caffeine may act as an ergogenic aid via non‐hypoalgesic mechanisms, studies have indicated that caffeine may also reduce the intensity of EIP during fixed intensity cycling exercise (e.g., Duncan et al., 2014). However, this hypoalgesic effect seems to only persist for mild to moderate intensities (≤3/10) of EIP (Black et al., 2015). During self‐paced exercise, no difference in EIP intensity is observed, despite greater average power outputs (Black et al., 2015). This could suggest that a greater power output can be produced for a given pain intensity, whereby individuals pace their time‐trials based on internal feedback in the form of EIP perceptions (Black et al., 2015). Taken together, caffeine consumed at a dose of 3–5 mg kg^−1^ body mass approximately 60 min prior to exercise may blunt perceptions of EIP and facilitate endurance exercise performance, particularly for lower intensity bouts of cycling. 

### 5.2 Transcutaneous electrical nerve stimulation

Transcutaneous electrical nerve stimulation (TENS) is a well‐established method for reducing chronic pain (Paley et al., 2021), but its efficacy for reducing EIP is less known. Astokorki and Mauger (2017b) provided evidence that TENS applied to the exercise musculature during an elbow flexion time‐to‐exhaustion task resulted in a 12% mean reduction in pain intensity, and a 38% longer endurance time. Furthermore, within the same study, lower‐limb application of TENS significantly improved cycling time‐trial performance by 2%. However, other work has reported no clear hypoalgesic or ergogenic effect of TENS (Hibbert et al., 2017), but these null findings could be explained by only using TENS prior to and not during exercise, like Astokorki and Mauger (2017b). One potential mechanism for an ergogenic effect of TENS may be due to the improved muscle blood flow and oxygenation (Tomasi et al., 2015). Theoretically, this could improve the clearance rate of noxious metabolites which are responsible for EIP. A more likely explanation is through the gate control theory of pain (Melzack and Wall, 1965), whereby stimulation of non‐nociceptive afferent nerves attenuates transmission of nociceptive signals at the spinal cord. In this context, the weak electrical current stimulates the Aβ fibres and inhibits C‐nerve fibres stimulated by intense exercise, resulting in a reduction in EIP. Taken together, TENS, or similar interventions which could improve blood flow and stimulate non‐nociceptive afferents may be promising methods to reduce the intensity of EIP during exercise and improve performance.

### 5.3 Cognitive strategies

One cognitive strategy that is featured heavily in the psychological literature is reappraisal, which involves individuals reforming their perceptions towards an experience (Lazarus, 1991) such as EIP. Reappraisal theory contends that individuals can reappraise EIP to elicit functional psychophysiological changes across the body that allow for better resource distribution (Jones et al., 2009). Lasnier and Durand‐Bush (2022) indicated endurance athletes would reappraise EIP by ‘accepting and committing to the pain’ or segmenting an exercise into more manageable chunks to ensure the EIP did not seem overwhelming. Neuroscientific studies concur that reappraisal also reduces the activation in the thalamus and other cerebral sites which are involved with the generation of perceptions of pain intensity (Moodie et al., 2020). Therefore, reappraisal may be an effective way to reduce EIP intensity during exercise and can therefore benefit exercise performance. However, there is a palpable lack of literature investigating the role of reappraisal on EIP indices and subsequent exercise performance.

Practitioners and athletes may wish to identify with other cognitive strategies such as dissociation from EIP. In a similar fashion to TENS, dissociation may cause less integration of nociceptive signals that contribute to EIP (Torta et al., 2017; van Damme et al., 2010). Several studies exhibit that an additional information source that draws attention (e.g., opponent, distraction) results in a lower EIP perception (Torta et al., 2017; Van Damme et al., 2010) or improved exercise performance (Williams et al., 2015). Yet, no study has merged the two to show that distraction during an exercise reduces EIP and potentially improves performance; however, there seems to be a reasonable basis of findings to suggest distractive strategies can improve exercise performance by changing perceptions of EIP.

## 6 FUTURE RESEARCH AND CONCLUSIONS

In summary, after defining EIP, the present review discussed the aetiology of EIP and its distinction from other exercise‐related pain (e.g., DOMS, injury). Accordingly, the review has related several existing pain induction models and how specific models (e.g., hypertonic saline) may be more appropriate when investigating the EIP experience due to their similarities in nociceptive stimulation and sensory processing pathways. As such, readers may be interested in using this review to provide operational and conceptual clarity to the EIP phenomenon and guide decisions on which a pain model is best suited to future experimental studies. Moreover, details of current pain measures such as the VAS and McGill pain scales and the potential to expand these methods to provide more concentrated measures of an individual's response to stimuli (e.g., quantitative sensory testing) are related. Successively, the article has discussed the *generally* negative effects EIP has on exercise performance and the literature supporting the neuro‐psychophysiological mechanisms of this effect. However, less is known about the effects of EIP on self‐paced exercise decisions or the possible effects of endogenous pain modulation during exercise tasks of varying intensities with concurrent pain stimulation. Furthermore, whilst this review has outlined potential interventions that can mitigate the generally negative effect of EIP on exercise performance, most interventions are founded on a limited body of research. Therefore, the field may benefit from future work that explores the EIP effect across more exercise task paradigms and validates the efficacy of the interventions highlighted within.

## AUTHOR CONTRIBUTIONS

Authors Callum A. O'Malley, Samuel A. Smith and Ryan Norbury contributed to the conceptualisation, writing and editing of the manuscript. Alexis R. Mauger contributed to the writing and editing of the manuscript. All authors have read and approved the final version of this manuscript and agree to be accountable for all aspects of the work in ensuring that questions related to the accuracy or integrity of any part of the work are appropriately investigated and resolved. All persons designated as authors qualify for authorship, and all those who qualify for authorship are listed.

## CONFLICT OF INTEREST

This paper generated no new data and authors report no conflicts of interest in the production of this manuscript.

## FUNDING INFORMATION

The publication of this manuscript was supported by the lead author's institutional agreement as part of the Wiley Read & Publish Transformative Agreement (JISC Gold CY24). No extra sources of funding were obtained to support the generation of this manuscript and its content.
